# Sex-specific cardiac dysfunction in mice with chronic kidney disease

**DOI:** 10.1093/ndt/gfaf056

**Published:** 2025-03-21

**Authors:** Yitong Zhao, Karen Yang, Christy M Nguyen, Hongmei Wu, Han Liu, Leandro M Velez, Jin Kyung Kim, Marcus Seldin, Wei Ling Lau

**Affiliations:** Division of Nephrology, Department of Medicine, University of California-Irvine, Irvine, CA, USA; School of Medicine, University of California-Riverside, Riverside, CA, USA; Department of Biological Chemistry, University of California-Irvine, Irvine, CA, USA; Division of Nephrology, Department of Medicine, University of California-Irvine, Irvine, CA, USA; Division of Nephrology, Department of Medicine, University of California-Irvine, Irvine, CA, USA; Department of Biological Chemistry, University of California-Irvine, Irvine, CA, USA; Division of Cardiology, Department of Medicine, University of California-Irvine, Irvine, CA, USA; Department of Biological Chemistry, University of California-Irvine, Irvine, CA, USA; Division of Nephrology, Department of Medicine, University of California-Irvine, Irvine, CA, USA

**Keywords:** cardiac function, chronic kidney disease, echocardiogram, mouse models

## Abstract

**Background:**

Cardiovascular disease (CVD) is the leading cause of death among patients with chronic kidney disease (CKD). Rodent models are widely used to study uremic CVD pathophysiology. We compared cardiac function parameters in male and female animals from two established mouse CKD models and examined associations between gut-derived uremic toxins and echocardiogram findings.

**Methods:**

Male and female adult C57Bl/6J mice were randomly assigned to control, adenine-induced CKD and 5/6 nephrectomy CKD groups. Echocardiography was performed on all mice at age 17 weeks (5 weeks after CKD induction). Serum creatinine, cystatin C and gut-derived uremic toxins were analyzed at study termination, and RNA sequencing of left ventricle tissue was performed and analyzed.

**Results:**

Markers of kidney dysfunction were elevated in both CKD models. The gut-derived uremic toxin indoxyl sulfate was increased in both CKD models, while trimethylamine N-oxide was increased in adenine CKD mice and p-cresyl sulfate in nephrectomy animals. Left ventricular volume was increased in nephrectomy animals. Cardiac output was decreased in male CKD animals from both models compared with controls, and ejection fraction was decreased in male 5/6 nephrectomy mice. Female controls had lower stroke volume and cardiac output than male counterparts, and female CKD animals had preserved cardiac output and ejection fraction when compared with female controls. The gut-derived uremic toxins trimethylamine N-oxide and indoxyl sulfate correlated with decreased cardiac output in male animals. Transcriptomics of cardiac tissue revealed sex-based variations in matrix metalloproteinase and mitochondrial pathways associated with cardiac dysfunction.

**Conclusions:**

Our work highlights sex differences in cardiac function and serum chemistries in two established preclinical CKD models. Gut-derived uremic toxins may impact cardiorenal pathophysiology and low cardiac output in male CKD animals.

KEY LEARNING POINTS

- Sex differences in echocardiographic parameters: the study revealed significant differences between male and female animals in echocardiographic measurements related to cardiac function in chronic kidney disease (CKD) models.
- Correlation of uremic toxins with cardiac parameters: the study found that the gut-derived uremic toxins trimethylamine N-oxide and indoxyl sulfate correlated with decreased cardiac output in male animals, whereas p-cresyl sulfate was associated with decreased ejection fraction in female mice, underscoring the importance of considering sex differences in the relationship between uremic toxins and cardiac function.
- Differential gene expression in CKD models: RNA sequencing of the left ventricle identified sex differences in terms of differentially expressed genes associated with cardiac dysfunction in CKD, particularly within matrix metalloproteinase and mitochondrial pathways.

## INTRODUCTION

Chronic kidney disease (CKD) is a global health problem with an estimated prevalence of 13% worldwide [1]. CKD results in many major health complications including cardiovascular disease (CVD), which is the leading cause of death worldwide. CKD patients are more likely to die of CVD before reaching end-stage renal disease, and experience a 10- to 30-fold increase in cardiovascular mortality compared with those without CKD [2]. Multiple mechanisms in the uremic milieu contribute to the development of CVD including endothelial dysfunction, chronic systemic inflammation, vascular calcification, and altered intestinal barrier and microbiota [3–6].

Two established rodent models used to study uremic CVD pathophysiology are the 5/6 nephrectomy and adenine-induced CKD mouse models [7]. Both models result in increased blood urea nitrogen and creatinine consistent with decreased glomerular filtration rate and decline in body weight. The 5/6 nephrectomy model mimics nephron loss with resultant proteinuria, hypertension and kidney histologic changes, including focal hypertrophy, glomerulosclerosis, tubular injury and interstitial fibrosis [8]. Increased systolic blood pressure and decreased cardiac systolic and diastolic function are also observed [8]. The nephrotoxic adenine model involves intratubular precipitation of insoluble 2,8-dihydroxyadenine crystals which lead to chronic tubulointerstitial injury, inflammation and fibrosis, as well as increased serum uric acid which induces cardiac and renal damage [9, 10]. Blood pressure is not typically elevated in the adenine CKD model [11], however rats on 0.25% adenine diet for 16 weeks have been reported to develop hypertension with left ventricular (LV) hypertrophy, increased cardiac collagen deposition and decreased aortic contractility [9, 12].

Gut-derived uremic toxins in the CKD milieu include p-cresyl sulfate (pCS), indoxyl sulfate (IS) and trimethylamine N-oxide (TMAO) which are produced from microbial metabolism of nutrients [4]. These toxins incur systemic inflammation and endothelial/vascular smooth muscle cell dysfunction, and increased blood levels are associated with higher risk of cardiovascular events and mortality in the CKD population [13, 14]. Animal CKD models have implicated gut-derived toxins in the pathogenesis of uremic cardiac dysfunction [15, 16], however echocardiogram parameters have not previously been studied across models and by sex. Echocardiography allows for non-invasive *in vivo* heart structure and function assessment using ultrasound. LV systolic function and remodeling are assessed by LV ejection fraction, cardiac output, ventricular size based on LV volume, and LV posterior wall (LVPW) thickness [17–19]. In this study, we compare echocardiogram correlates of cardiac function and evaluate heart RNA transcriptomics in male and female C57BL/6J mice following CKD induction via two models: dietary adenine-induced and 5/6 nephrectomy. Furthermore, we examine associations between gut-derived uremic toxins and echocardiographic parameters to investigate the kidney–gut–heart axis.

## MATERIALS AND METHODS

### Animal models

**Figure 1:**
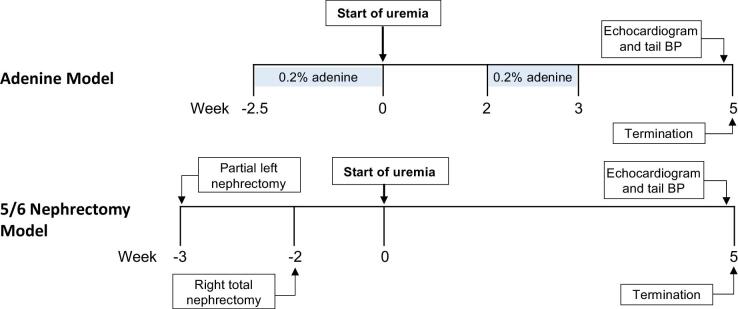
Experimental timeline, two CKD mouse models. In the adenine-CKD model, mice were fed a 0.2% adenine diet for 18 days followed by 2 weeks on regular chow. Mice were then re-exposed to 0.2% adenine diet to sustain CKD. For the 5/6 nephrectomy model, age-matched male and female nephrectomized mice were purchased from the Jackson Laboratory. Echocardiography and tail blood pressure measurements were performed 5 weeks after uremia induction, prior to study termination. Serum was collected to measure kidney and gut-derived toxin biomarkers, and heart tissues were collected for RNA analysis.

All animal studies were approved by the Animal Research Committee, University of California, Irvine (UCI) under protocol AUP-21-157. Animals were maintained at the UCI vivarium in accordance with the policies instituted by the American Association for Accreditation of Laboratory Animal Care. Male and female C57Bl/6J mice aged between 8 and 10 weeks were purchased from the Jackson Laboratory (Bar Harbor, ME, USA) and were randomly assigned to control vs adenine-induced CKD. Mice in adenine groups were fed a 0.2% adenine diet for 18 days, followed by 2 weeks on regular chow and then a second 1-week period on adenine diet to maintain CKD (Fig. 1). Age-matched male and female 5/6 nephrectomized mice were purchased from the Jackson Laboratory. The initiation timepoint of uremia was considered to be after 18 days of adenine diet (adenine-CKD model) or 14 days post-surgery (5/6 nephrectomy model). Echocardiography was performed at 5 weeks after uremia induction at age 18 weeks (*n* = 8 per group). Tail blood pressure was measured prior to study termination via tail-cuff plethysmography which included 10 habituation cycles followed by 10 measurements, with automated exclusion of outlier values (CODA-S2 multi-channel, Kent Scientific). Body weight was monitored weekly to assess the overall health of the mice and to track the progression of kidney disease, and body weight at termination is presented in Table 1. At study termination, blood was collected via cardiac puncture and processed for serum collection. Heart tissue was collected for RNA analysis.

**Table 1:** Serum chemistries.

	Male			Female		
	Control (*n* = 8)	Adenine (*n* = 8)	5/6 nephrectomy	Control (*n* = 8)	Adenine (*n* = 8)	5/6 nephrectomy
Body weight at termination (g)	29.50 ± 0.63	23.63 ± 0.57^a^	26.71 ± 0.52^a,b^	21.00 ± 0.33^c^	19.25 ± 0.41^a,c^	22.29 ± 0.29^b,c^
Serum creatinine (mg/dL)	0.05 ± 0.003	0.24 ± 0.02^a^	0.11 ± 0.01^a,b^	0.07 ± 0.01	0.16 ± 0.02^a,c^	0.16 ± 0.01^a^
Cystatin C (mg/L)	0.14 ± 0.1	0.93 ± 0.09^a^	0.35 ± 0.06^a,b^	0.09 ± 0.02	0.45 ± 0.05	0.30 ± 0.05^a^
p-Cresyl sulfate (µM)	2.7 ± 0.5	4.2 ± 1.1	9.5 ± 3.7^a^	0.7 ± 0.5	1.4 ± 0.5	14.8 ± 2.5^a,b^
Indoxyl sulfate (µM)	2.1 ± 0.3	19.7 ± 5.0^a^	8.5 ± 1.0	7.0 ± 1.2	23.7 ± 4.1^a^	28.2 ± 4.8^a,c^
Trimethylamine-N-oxide (µM)	0.04 ± 0.1	15.4 ± 3.5^a^	0.6 ± 0.2^b^	2.5 ± 0.6	9.3 ± 2.0^a^	8.3 ± 2.0^c^

^a^*P* < .05 compared with control mice of same sex.

^b^*P* < .05 compared with adenine CKD mice of same sex.

^c^*P* < .05 significant sex differences within same model.

### Echocardiography

**Figure 2:**
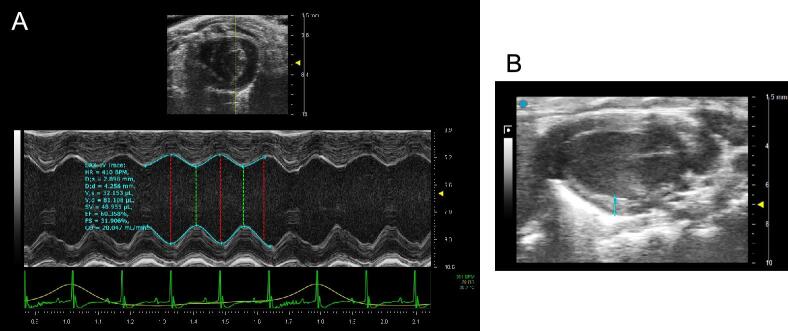
Settings on the Vevo 3100 ultrasound system. (**A**) LV parameters were calculated from the parasternal short-axis M-mode view, using manual tracing at the level of two papillary muscles. (**B**) LVPW thickness during diastole was measured on B-mode long-axis view by tracing the LVPW at the midpoint of the heart.

Echocardiography was performed at 5 weeks after uremia induction, at age 18 weeks (Fig. 1). After anesthesia induction with isoflurane, animals were maintained under inhaled 1.5% isoflurane and 95% O_2_ to allow for spontaneous breathing [20, 21]. Animals were imaged in a left lateral decubitus position and warming pads were used to maintain normothermia [21]. Images of the horizontal long-axis view and short-axis view of the heart were collected in both M-mode and B-mode [20] using a Vevo 3100 ultrasound system with 25-mHz scan head (VisualSonics, Toronto, ON, Canada). The “LV trace” feature was used to calculate LV structural and functional parameters from the LV parasternal short-axis M-mode view, which was recorded at the level of two papillary muscles (Fig. 2A) [22]. LVPW thickness during diastole was measured on B-mode long-axis view (Fig. 2B). Vevo analysis software (VevoLab, VisualSonics) was used to conduct all measurements and calculations. Vevo software analysis was performed by a research team member blinded to study groups (H.W.).

### Serum chemistries

Serum creatinine collected at study termination was analyzed via liquid chromatography–mass spectrometry at the O'Brien Center, University of Alabama at Birmingham (Birmingham, AL, USA). Cystatin C was measured using the Invitrogen Cystatin C (CST3) Mouse ELISA Kit (EMCST3, Fisher Scientific). Total serum levels (free and protein-bound) of the gut-derived uremic toxins pCS, IS and TMAO were measured using liquid chromatography with tandem mass spectrometry (HPLC-MS/MS) based on previously described methods [23, 24]. A 25-μL aliquot of serum was treated with 200 μL of acetonitrile with 0.1% formic acid and internal standard for protein precipitation. The mixture was vortexed and centrifuged, and evaporated to dryness. The dried extract was reconstituted with 100 μL of 25% acetonitrile. Standards and prepared samples were injected (10 μL) into the HPLC-MS/MS instrument, Waters Quattro Premier XE equipped with UPLC. For IS and pCS, hydrochlorothiazide was used as the internal standard and analysis was performed in negative ionization mode. For TMAO, salbutamol was used as the internal standard and analysis was performed in positive ionization mode [25].

### RNA sequencing

Total RNA was isolated from LV heart tissue using TRIzol^®^ reagent (Life Technologies, Carlsbad, CA, USA), followed by an additional DNase I digestion to remove genomic DNA contamination. RNA samples were submitted to the UC Irvine Genomics Research and Technology Hub (GRTH) for sequencing with the NovaSeq 6000 platform S4 (Illumina, San Diego, CA, USA). RNA quality was verified using the Agilent Bioanalyzer Nano RNA chip (Agilent Technologies, Santa Clara, CA, USA) and Nanodrop (Thermo Fisher Scientific, Waltham, MA, USA). Samples in the current study showed A260/A280 absorbance ratios in the range 1.928–2.236. Library construction was performed according to the Illumina TruSeq mRNA stranded protocol using the Apollo 324 library prep system (Takara Bio, Mountain View, CA, USA). The input quantity for total RNA was 1 µg and mRNA was enriched using oligo-dT magnetic beads. The enriched mRNA was chemically fragmented for 3 min. First-strand synthesis used random primers and reverse transcriptase to make cDNA. After second-strand synthesis, the cDNA was cleaned using AMPure XP beads, followed by end repair and 3′ adenylation. Illumina dual-indexed adapters were ligated on the ends and the adapter-ligated fragments were enriched by nine cycles of PCR. The resulting libraries were validated by qPCR (KAPA Library quantification kit, Kapa Biosystems, Wilmington, MA, USA) and sized with the Agilent Bioanalyzer DNA high-sensitivity chip. Library concentrations were normalized and then multiplexed together. The multiplexed libraries were sequenced using paired-end with read length 150 base pairs. Fastq files obtained from sequencing were inspected via FastQC then aligned to the current mouse genome build (mm10) using STAR. Counts matrices were then generated from aligned BAM files using summarizeOverlaps (Genomicfeatures) and differential expression was performed using DESeq2 with false discovery rate (FDR) adjusted *P*-value <.001 [26]. All plots were generated in R. Pathways derived from differentially expressed genes (DEGs) were identified using Gene Set Enrichment Analyses based on erichR focusing queries on Reactome, Gene Ontology and Kyoto Encyclopedia of Genes and Genomes (KEGG) databases [27].

### Statistical analysis

Data are presented as the mean ± standard error of the mean. Outliers were excluded using the ROUT test with Q = 1%. Two-way analysis of variance (ANOVA) with Tukey's multiple comparisons test were performed using GraphPad Prism 8.4 (GraphPad Software, San Diego, CA, USA). Šídák's multiple comparisons test was used to determine sex differences. Blood chemistries did not follow a Gaussian distribution per the Kolmogorov–Smirnov normality test, therefore nonparametric Spearman correlation analysis was used to analyze associations between toxin levels and echocardiographic parameters. Linear regression analysis was computed including r slope and *P*-values. *P*-values <.05 were considered statistically significant.

## RESULTS

### Uremic toxins: CKD model and sex differences

**Figure 3:**
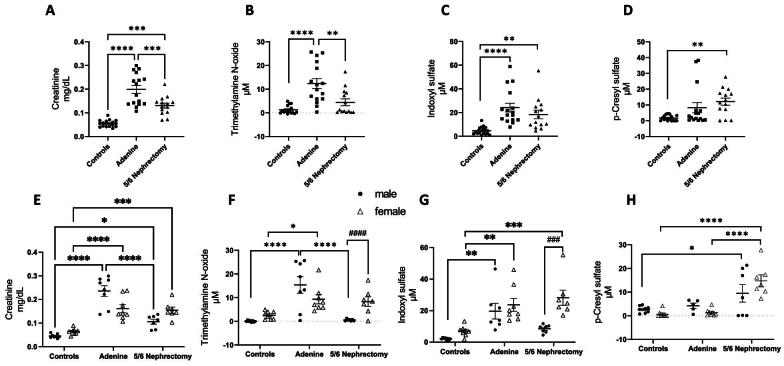
Uremic toxin levels were variably increased in two mouse CKD models. Levels of serum creatinine (**A**) and the gut-derived toxins TMAO (**B**), IS (**C**) and pCS (**D**) were elevated in CKD models globally. Levels of uremic toxins stratified by sex are shown in (**E**–**H**). *P < .05, **P < .01, ***P < .001, ****P < .0001, ^###^*P* < .001 and ^####^*P* < .0001 denote sex differences within CKD model.

To compare kidney function markers and levels of blood uremic toxins between the CKD models, serum creatinine and cystatin C, as well as levels of the gut-derived uremic toxins were measured and are summarized in Table 1. Body weight at termination was significantly lower in both CKD models in both sexes. Females consistently had lower body weights compared with males. At 5 weeks post-uremia induction, serum creatinine and cystatin C were significantly elevated in both CKD models. The same trends were observed when creatinine and cystatin C were adjusted for body weight (Supplementary data, Table S1). The gut-derived uremic toxins pCS, IS and TMAO were variably increased in the CKD models compared with control animals (Fig. 3). When analyzed by model (before stratification by sex), TMAO was significantly increased in the adenine CKD group (Fig. 3B), IS was elevated in both CKD models (Fig. 3C) and pCS was increased in the nephrectomy group. When stratified by sex, female nephrectomized animals had significantly higher serum TMAO and IS levels compared with male counterparts (Fig. 3F and G).

### Cardiac dysfunction in CKD mouse models

**Figure 4:**
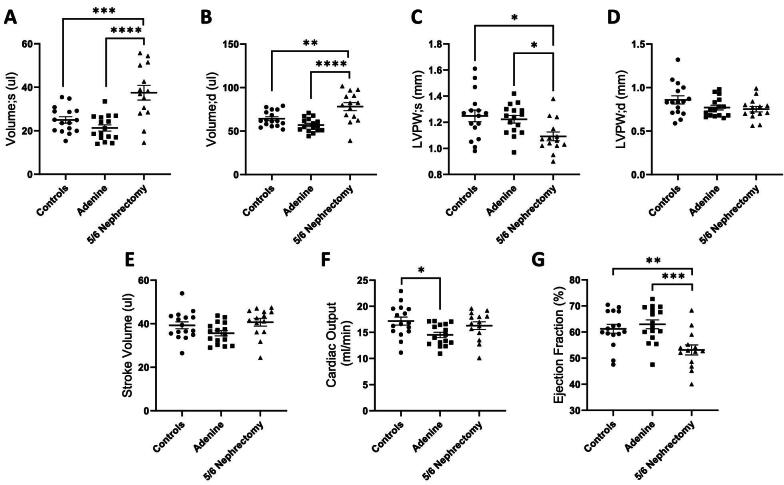
Cardiac dysfunction in adenine-CKD and nephrectomy-CKD mouse models (grouped male and female data). Cardiac parameters including LV volume during systole (**A**) and diastole (**B**), LVPW thickness during systole (**C**) and diastole (**D**), SV (**E**), CO (**F**) and EF (**G**) were analyzed using the Vevo echo ultrasound system. *P < .05, **P < .01, ***P < .001, ****P < .0001.

**Figure 5:**
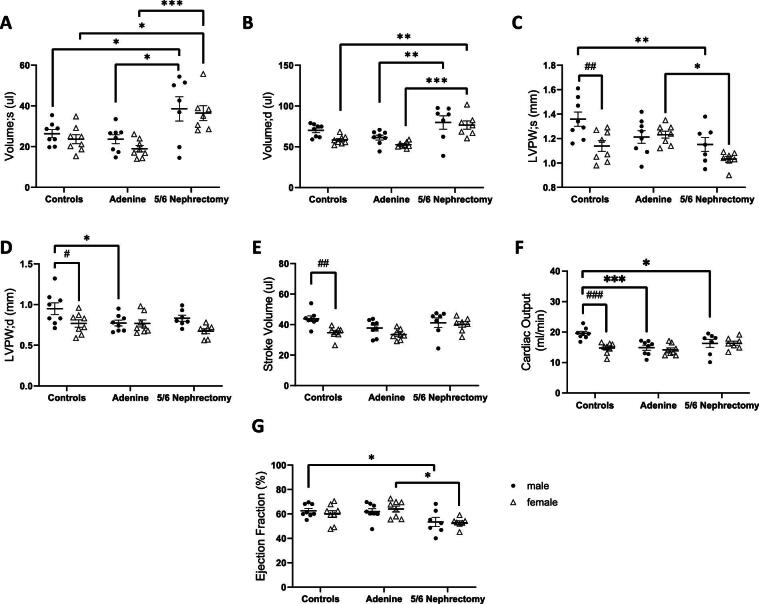
Cardiac parameters stratified by sex. LV volume during systole (**A**) and diastole (**B**), LVPW thickness during systole (**C**) and diastole (**D**), SV (**E**), CO (**F**) and EF (**G**). *P < .05, **P < .01, ***P < .001, ^#^*P* < .05 sex difference, ^##^*P* < .01 sex difference, ^###^*P* < .001 sex difference.

At 5 weeks following uremia induction, echocardiography was done to evaluate cardiac function in the two CKD models. Blood pressure and echocardiogram parameters are summarized in Table 2 and are further presented by CKD model (Fig. 4) and stratified by sex (Fig. 5). Systolic blood pressure in male 5/6 nephrectomy mice trended higher compared with male controls (*P* = .07). LV volume during systole and diastole (Volume; s in Fig. 4A and Volume; d in Fig. 4B) were significantly increased while LVPW thickness in systole was significantly reduced (Fig. 4D) in the nephrectomy group, but not in adenine-induced CKD mice. The stroke volume was not altered (SV, Fig. 4E). Adenine-induced CKD mice demonstrated decreased cardiac output (CO, Fig. 4F) while ejection fraction (EF) was significantly reduced in the nephrectomy group (Fig. 4G).

**Table 2:** Blood pressure and echocardiogram parameters of cardiac function in CKD mice.

	Male			Female		
	Control	Adenine	5/6 nephrectomy	Control	Adenine	5/6 nephrectomy
	(*n* = 8)	(*n* = 8)	(*n* = 7)	(*n* = 8)	(*n* = 8)	(*n* = 7)
Heart rate (bpm)	449 ± 13	394 ± 11	397 ± 18	426 ± 9	422 ± 7	408 ± 6
Systolic blood pressure (mmHg)	122 ± 5	122 ± 4	135 ± 5	135 ± 4	123 ± 4	127 ± 7
Diastolic blood pressure (mmHg)	91 ± 6	95 ± 4	102 ± 5	101 ± 5	88 ± 4	90 ± 7
LVPW in systole (mm)	1.36 ± 0.06^a^	1.21 ± 0.05	1.15 ± 0.06^a^	1.14 ± 0.04^c^	1.23 ± 0.03	1.03 ± 0.02^b^
LVPW in diastole (mm)	0.95 ± 0.07^c^	0.77 ± 0.04	0.83 ± 0.04 ^a^	0.77 ± 0.05^c^	0.77 ± 0.04	0.67 ± 0.03
LV volume in systole (µL)	26.6 ± 0.8	25.4 ± 1.0	30.6 ± 2.2 ^a,b^	25.4 ± 1.0	23.3 ± 0.8	30.3 ± 1.2 ^a,b^
LV volume in diastole (µL)	40.0 ± 0.7	37.8 ± 0.8	41.9 ± 2.0^b^	37.0 ± 0.5	35.5 ± 0.4	41.4 ± 1.2^a,b^
Stroke volume (µL)	43.8 ± 1.8^c^	37.7 ± 1.9	41.2 ± 3.2	34.7 ± 1.4^c^	33.5 ± 1.2	40.1 ± 1.8
Cardiac output (mL/min)	19.6 ± 0.7^c^	14.9 ± 0.8^a^	16.3 ± 1.3^a^	14.8 ± 0.6^c^	14.2 ± 0.6	16.3 ± 0.7
Ejection Fraction (%)	63 ± 1.9	62 ± 2.5	53 ± 3.6^a^	60 ± 2.9	64 ± 2.4	53 ± 1.5^b^

^a^*P* < .05 compared with control mice of same sex.

^b^*P* < .05 compared with adenine CKD mice of same sex.

^c^*P* < .05 significant sex differences within same model.

When stratified by sex, male and female nephrectomized mice demonstrated increased LV volume compared with adenine CKD counterparts (Fig. 5A and B). LVPW thickness in end-systole and end-diastole was lower in female vs male control mice (Fig. 5C and D). Compared with male controls, male CKD animals from both models had decreased LVPW thickness. Female nephrectomized animals had decreased LVPW thickness compared with adenine counterparts, however the two female CKD cohorts were not significantly different from female controls (Figs. 5C and D). Female controls had significantly lower SV than male controls (Fig. 5E), and CO was significantly decreased in males from both CKD models compared with controls (Fig. 5F). Low EF was noted in the nephrectomized mice; this was significantly decreased in the male nephrectomy group compared with sex-matched controls, and in the female nephrectomy group compared with female adenine CKD mice (Fig. 5G).

### Gut-derived uremic toxins and cardiac parameters

**Figure 6:**
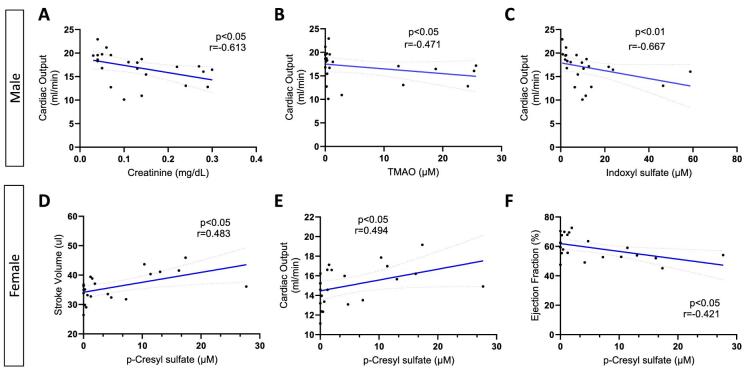
Correlations between kidney function and uremic toxin levels with cardiac parameters. When stratified by sex, creatinine (**A**), TMAO (**B**) and IS (**C**) were negatively correlated with CO in male mice. Conversely, female mice showed significant correlations between pCS and increased SV (**D**) and CO (**E**). pCS was associated with lower EF in female mice (**F**) but not in male animals.

To investigate the association between kidney function and gut-derived uremic toxins with cardiac function, Spearman correlation analyses were performed, and findings are summarized in Supplementary data, Fig. S1. The kidney function markers creatinine and cystatin C were positively correlated with the gut-derived toxins TMAO, IS and pCS. When stratified by sex, male animals showed significant associations between creatinine (Fig. 6A), cystatin C, TMAO (Fig. 6B) and IS (Fig. 6C) with lower CO. Conversely, female animals showed significant correlations between pCS levels with increased SV (Fig. 6D) and CO (Fig. 6E), and decreased EF (Fig. 6F). The associations were unchanged when correlation analyses were done with weight-adjusted creatinine and cystatin C (data not shown).

### Heart RNA sequencing

**Figure 7:**
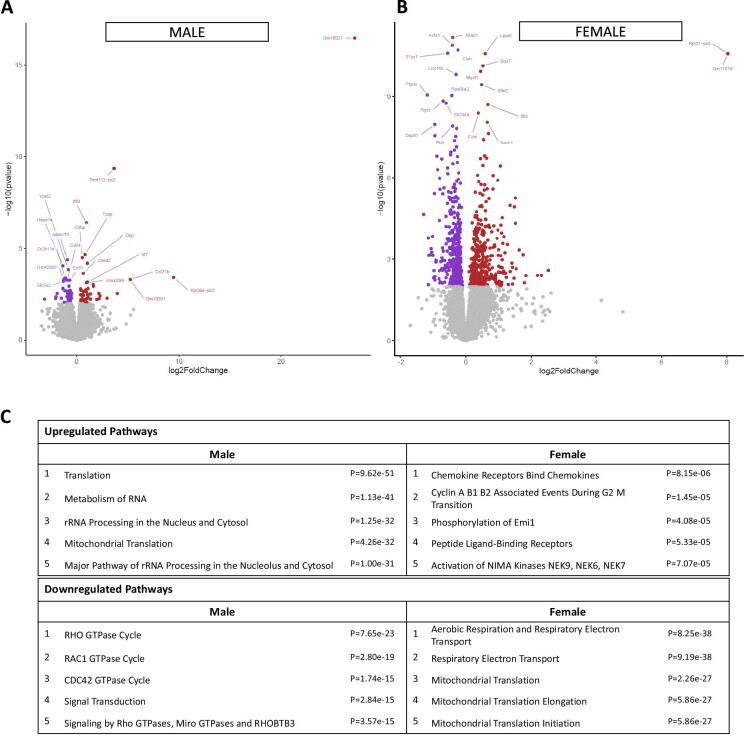
Heart RNA sequencing and enrichment analysis. (**A**) LV total RNA sequencing in male CKD mice (adenine-CKD and 5/6 nephrectomy mice, *n* = 8) vs male controls (*n* = 4) showed increased transcripts for Gm10221 (long non-coding RNA), with log2 fold-change 27.201, FDR-adjusted *P* < .001. Rpl36a-ps2 (involved in ribosome biogenesis) and Trmt112-ps2 (methyltransferase involved in cardiomyocyte hypertrophy) were also upregulated (log2 fold-change 9.47 and 3.65, respectively, *P* < .001 for both genes). (**B**) LV total RNA sequencing in female CKD mice (adenine-CKD and 5/6 nephrectomy mice, *n* = 8) vs female controls (*n* = 4) demonstrated increased transcripts for Rpl27-ps3 (ribosomal protein L27 pseudogene 3), with log2 fold-change 8.04 and adjusted *P* < .001. (**C**) Enrichment pathways analyzed by Reactome, Gene Ontology and KEGG databases identified the top five upregulated and downregulated pathways in CKD stratified by male and female mice.

To evaluate transcriptomic pathways underlying the LVPW thickness and CO differences observed in CKD groups, RNA sequencing analysis of cardiac (left ventricle) tissue was done from both male and female mice across three experimental groups: controls, adenine CKD and 5/6 nephrectomy (*n* = 4 per group). Using DESeq2 analysis we identified the top 20 DEGs in males and females (Fig. 7A and B). There were 14 upregulated and 6 downregulated genes in male CKD animals compared with controls. Genes involved in ribosome biogenesis (*Rpl36a-ps2*) [28] and cardiac hypertrophy (*Trmt112-ps2*) [29] were upregulated, whereas genes involved in cardiovascular morphogenesis (*Adam19*) [30], cardiac repair (*Hspa1a*) [31] and cellular trafficking (*Ypel2*) [32] were downregulated. Similarly, in female CKD animals, genes involved in ribosome biogenesis (*Rpl27-ps3*) [28] and development of atherothrombotic vascular disease (*Lpar6*) [33] were upregulated; in contrast, genes contributing to signal transduction pathways (*Akap1*) [34] and lipid metabolism (*Cluh*) [35] were downregulated (Supplementary data, Fig. S2).

Upon evaluation of broad metabolic pathways using erichR focusing queries on Reactome, Gene Ontology and KEGG databases, differential expression analysis revealed notable findings in CKD mice by sex (Fig. 7C). In CKD males, RNA metabolism and mitochondrial translation were significantly upregulated, while in CKD females there was upregulation of chemokine receptors, phosphorylation of Emi1 and peptide ligand-binding receptors. Conversely, the top five downregulated pathways in CKD males included signal transduction by Rho GTPases, micro GTPases and RHOBTB3. In females, downregulated pathways in CKD included respiratory electron transport and mitochondrial translation/initiation (Fig. 7C).

## DISCUSSION

In this echocardiography study of two well-established mouse CKD models, we noted cardiac dysfunction differences at 5 weeks following induction of uremia by 5/6 nephrectomy vs adenine nephrotoxic diet. In male experimental groups, 5/6 nephrectomy mice manifested increased LV volume and decreased EF. There was likely a blood pressure effect on LV remodeling, as tail systolic blood pressure was on average 13 mmHg higher in the male nephrectomy mice, consistent with our prior published work [11]. CO was decreased in male CKD animals from both models. Male controls had significantly higher SV and CO than female controls; this sex difference was not apparent in the CKD groups due to cardiac dysfunction in the male CKD animals. In the two female CKD models, LVPW thickness, CO and EF were not significantly different from female controls (Table 2 and Fig. 5). Female nephrectomized animals had significantly higher serum TMAO and IS levels compared with males, however these toxins did not correlate with cardiac parameters in females. TMAO and IS toxins correlated with decreased CO in males, whereas pCS was associated with decreased EF in females (Fig. 6). Additionally, we observed that alterations in gene expression pathways varied in male and female heart tissue. RNA sequencing in male CKD mice showed upregulation of genes related to cardiac hypertrophy and downregulation of pathways involved with RNA metabolism, mitochondrial translation and rRNA processing in the nucleolus and cytosol. Transcriptomics in female CKD mice revealed downregulation of mitochondrial translation and respiratory electron transport.

Gut-derived uremic toxins incur systemic inflammation and vascular dysfunction [36, 37] and are associated with increased CVD and mortality risk in the CKD population [13, 14]. In the current study, serum levels of gut-derived uremic toxins varied by CKD model and sex. Male and female adenine CKD animals had increased levels of TMAO and IS compared with controls, while pCS was increased in nephrectomy CKD animals. Female nephrectomy mice demonstrated higher TMAO and IS levels compared with male nephrectomy counterparts. Interestingly, although CKD was most severe in adenine CKD males (which had the highest creatinine and cystatin C levels), IS and pCS were highest in nephrectomy CKD females (Fig. 3). This suggests differences in gut dysbiosis across CKD models, which requires further study. TMAO is a well-documented vascular toxin [38, 39] and correlated with decreased CO in males (r = –0.471, *P* < .05). In female animals, pCS was positively correlated SV and CO and negatively associated with EF (r = –0.421, *P* < .05), suggesting a potential role for pCS in heart failure with preserved EF [40]. Work by others has demonstrated that pCS and IS affect cardiac function along shared pathways including upregulating collagen fibrosis [41, 42] and increasing oxidative stress [43]. Our findings highlight sex differences in the microbiota-cardiovascular axis which has been described in emerging clinical data [44, 45].

The observed EF reduction in male 5/6 nephrectomy mice is consistent with the report by Hamzaoui *et al*. and suggests LV systolic dysfunction [8]. In contrast, Chen *et al*. reported LV hypertrophy in male nephrectomized CKD mice at 8 weeks post-surgery without EF change [46]. A recent comprehensive review of murine models of uremic cardiomyopathy discussed the challenges in assessing cardiac dysfunction in the 5/6 nephrectomy model; increased blood pressure, cardiac hypertrophy and systolic dysfunction were reported in only 40%–50% of studies [47]. Longer-term studies after 12 weeks of uremia revealed more consistent changes in terms of increased LV diameter, impaired EF and cardiac fibrosis [47]. Similarly, male rats with adenine-induced CKD were reported to manifest LV hypertrophy, decreased LV volume and decreased SV at 16 weeks post-uremia induction [9]. In the current study we noted decreased LVPW thickness in male CKD mice compared with controls; we hypothesize that the relatively short experimental timeline (5 weeks post-uremia induction) preceded development of LV hypertrophy.

In the current study, female mice in both adenine CKD and 5/6 nephrectomy models showed preserved CO and EF at 5 weeks after uremia induction (not significantly different from female controls), likely due to the cardio- and reno-protective effects of estrogen. Prior reports have demonstrated that female rodents are protected against cardiac and renal disease compared with their age-matched male counterparts [48, 49]. Kasimay *et al*. reported more severe kidney dysfunction in ovariectomized 5/6 nephrectomized female rats than in nephrectomized female rats with intact ovaries; administration of estradiol ameliorated kidney injury [49]. Estrogen therapy reduced levels of malondialdehyde, glutathione and myeloperoxidase in the heart, suggesting anti-inflammatory and antioxidant properties of estrogen in CKD-associated CVD [49]. Furthermore, agonists of estrogen signaling have been shown to directly regulate neo-angiogenesis, endothelial barrier maintenance and macrophage recruitment following cardiac injury [50]. In our study, male adenine CKD mice had the most severe kidney injury (highest creatinine and cystatin C levels) and these markers of kidney dysfunction were associated with lower CO only in males.

In total RNA sequencing analysis of LV tissue from the two CKD models (adenine CKD and 5/6 nephrectomy), the top 20 DEGs were identified that were significantly different from control hearts in each sex (Fig. 7). In CKD males, after FDR adjustment, significant downregulation of Adam19 (a disintegrin and metalloproteinase domain-containing protein 19) was noted. Adam19 is highly expressed in the endocardial cushion and conotruncus during embryonic heart development; mice lacking functional *Adam19* present disrupted smooth muscle cell ensheathment and ventricular septal defects [30]. Further studies are needed to elucidate the role of Adam19 in uremic cardiomyopathy. In CKD females, *Akap1* (a-kinase anchoring protein 1) and *Cluh* (clustered mitochondria homolog) were the top two downregulated genes, and both play important roles in cardiac function. One of the key roles of *Akaps* in the heart is the regulation of cardiac contractility. *Akaps* contribute to PKA signaling to regulate calcium-handling proteins such as RyR2 and phospholamban, which in turn influence cardiac contractility and the response of the heart to electrical signals [34]. The *Cluh* deficiency contributes to mitochondrial dysfunction, which is commonly observed in hypertrophic cardiomyopathy and heart failure [51].

Pathway enrichment analysis using the Reactome, Gene Ontology and KEGG databases highlighted sex differences in cardiac signaling pathways. Interestingly, mitochondrial translation was upregulated in males but downregulated in females. These differences are likely influenced by hormonal factors such as estrogen and testosterone, which contribute to sex-based variations in cardiac function [52]. Estrogen has been shown to enhance mitochondrial neogenesis, promote mitochondrial protein synthesis and improve mitochondrial function in the heart. Levels of estrogen decrease as CKD progresses, which contributes to mitochondrial dysfunction and further influences cardiac function [53]. The most significantly downregulated pathway in CKD male animals was the GTPase cycle, which is a fundamental process that regulates multiple aspects of cardiac function, including contractility, signal transduction and cellular maintenance. Reduction of GTPase activity is linked with hypertrophy and heart failure [54].

In conclusion, our work highlights sex differences in echocardiographic parameters and serum uremic toxin levels in two established preclinical CKD models. Decreased CO was observed in male CKD animals but not in females. The gut-derived toxins TMAO and IS were correlated with CO in males, whereas pCS correlated with EF in female animals. Pathway enrichment RNA analysis of heart tissue identified reduced GTPase activity in CKD males, whereas suppressed mitochondrial synthesis was prominent in CKD females. Further studies are needed to elucidate mechanisms by which gut-derived toxins may modulate these metabolic pathways. Our findings emphasize the importance of analyzing sex differences in studies and drug development related to cardiorenal pathophysiology.

## Supplementary Material

gfaf056_Supplemental_File

## Data Availability

The data underlying this article are available in the article and in its online supplementary material. Individual animal data and RNA-sequencing dataset available on Dryad: doi:10.5061/dryad.zcrjdfnps.
