# Supplementary material for: Sex-specific cardiac dysfunction in mice with chronic kidney disease
Source: Nephrol Dial Transplant. 2025 Mar 21;40(10):1865–75. doi: 10.1093/ndt/gfaf056 (PMC12548047; doi:10.1093/ndt/gfaf056)
Supplement: gfaf056_Supplemental_File [file gfaf056_Supplemental_File.docx]

Supplemental Data

**Table S1. Creatinine and cystatin C adjusted for body weight.**

Data are mean ± SEM

a = p < 0.05 compared to control mice of same sex; b = p < 0.05 compared to adenine CKD mice of same sex;

* p < 0.05 significant sex differences within same model

**Figure S1. Correlation analysis of serum toxins and echocardiogram parameters.** Nonparametric Spearman correlation analysis was done as blood toxin levels did not follow a Gaussian distribution per the Kolmogorov-Smirnov normality test. Data was analyzed using the entire dataset, and then stratified by male and female animals. n.s. = not significant


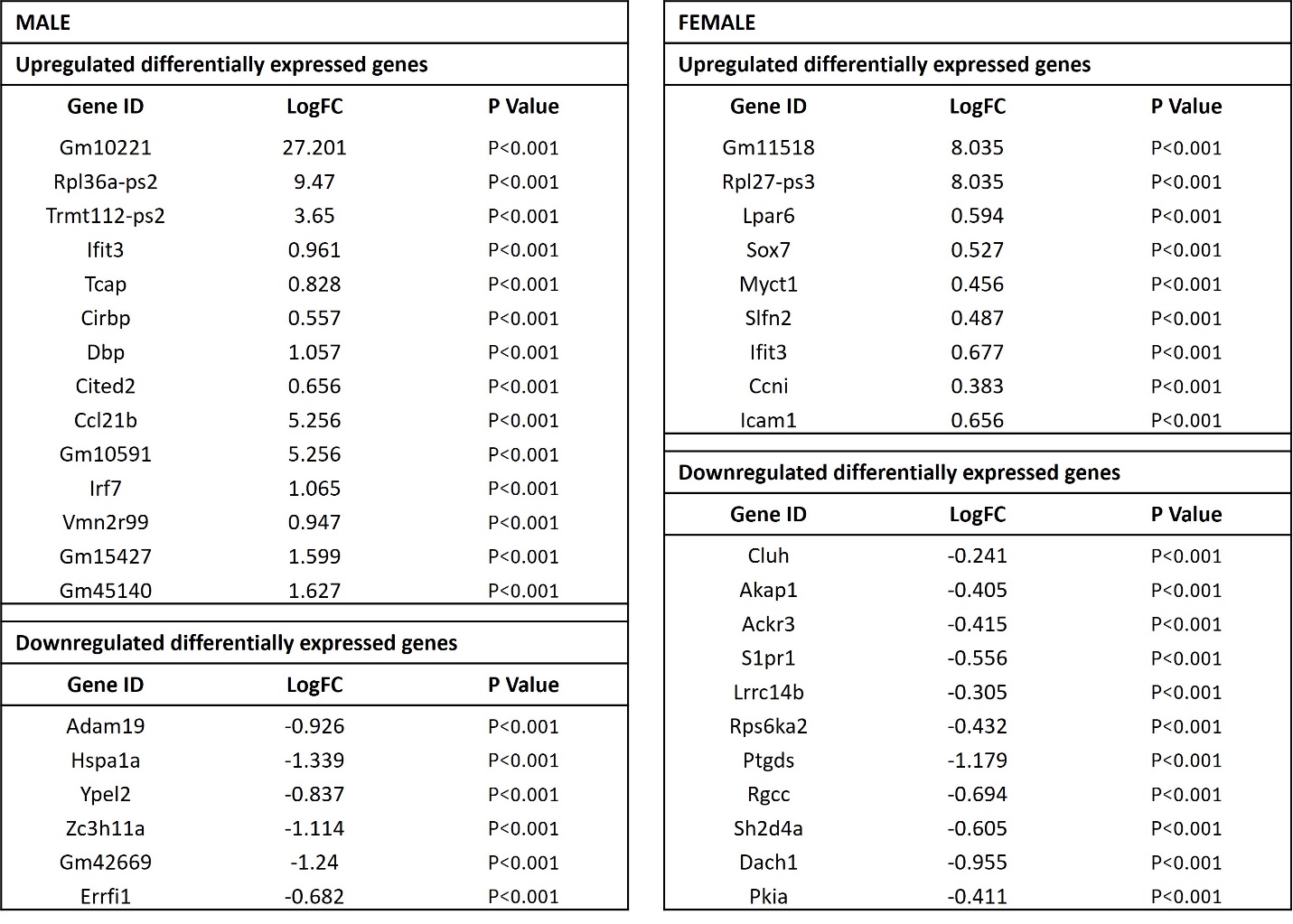


**Figure S2. Top twenty differentially expressed genes identified from heart samples of male and female mice.** Using DESeq2 analysis, the top twenty differentially expressed genes (DEGs) by sex were identified, comprising 14 upregulated genes and 6 downregulated genes in males, and 9 upregulated gene and 11 downregulated genes in females.
